# From the President’s Desk: Part 1, 2024

**DOI:** 10.4102/safp.v66i1.5900

**Published:** 2024-02-29

**Authors:** Andrew Ross

**Affiliations:** 1 Department of Family Medicine, Faculty of Medicine, University of KwaZulu-Natal, Durban, South Africa; 2 President, South African Academy of Family Practitioners, Durbanville, South Africa

2024 … a new year with new challenges and new opportunities.

In 2023, a new South African Academy of Family Physicians (SAAFP) Council and EXCO were elected, and we are committed to serving *you* as our members. At the start of a new term of office, we do want to acknowledge and thank Prof. Mash and the previous Council for the role that they have played in increasing the visibility and profile of the Academy, supporting education and training, developing continuing professional development (CPD) programmes and activities and strongly advocating for the critical role of family medicine in South Africa in both the public and private sectors.

As the new Council, we recognise that the strength of any organisation lies in its membership. We cannot meet the challenges facing the South African health sectors alone – we need *you* to actively participate, contribute and take on roles and responsibilities within the organisation and for you to send us your thoughts, ideas and suggestions. SAAFP is *our* organisation – to borrow from the recent rugby world cup – *Together we are stronger*. It is now possible to pay your membership as a monthly debit order (only R170.00/month × 10 months), and I would encourage everyone to use this payment method.

In October 2023, we had a very productive strategic planning meeting. Priorities for the new term of office include:

- Continuing to provide appropriate training and development opportunities to our members through:
  ■ Access to quality CPD activities through CPD articles, monthly webinars and short courses.
  ■ The SAAFP annual conference. The conference provides a wonderful opportunity to connect with colleagues, present your research and get all your CPD points. We had an excellent conference at the Wanderers in Johannesburg in August 2023 (thanks to Dr K. Naidoo and her team). The 2024 conference will be held at the Breakwater Lodge in Cape Town from 07 to 08 September 2024. Please put this in your diary, submit an abstract, plan to be there and actively participate. Details are on the SAAFP website: https://saafp.org/conferences/2024-conference/.
  ■ Point of Care Ultrasound (PoCUS) training. The Academy has access to the GUSI (Global Ultrasound Institute) online training material, and in the new year, we are planning practical training programmes in each of the major metropoles to help family physicians develop proficiency in the use of this excellent tool.
- Ongoing mentoring support for new family physicians through the Next 5 initiative.
- Continuing support for the *South African Family Practice* journal (SAFPJ), the official journal of the Academy, which has a Cite score of 1.1 and has recently been accepted onto the SciELO SA platform. Our thanks to Prof. K. von Pressentin and his colleagues for the leadership provided as the journal continues to provide an excellent platform to share research findings, CPD, Mastering your Fellowship as well as support for new graduates.
- Education and training committee (ETC), which continues to provide support for undergraduate and postgraduate training in family medicine.
- Advocacy:
  ■ Dr S. Matthew is leading the initiative for family physician (FP) in private to be recognised as a specialist by the medical aids to enable them to investigate, prescribe and be remunerated as specialists. SAAFP has partnered with Health Man, who will provide technical support to the Academy. Remember, together, we are stronger, and we encourage all members in private practice to join this initiative. Join, participate and get involved. Your voice needs to be heard.
  ■ Dr J. Nash has been appointed as the national spokesperson for the Academy. The position paper on the role of the family physician has been presented at national and provincial forums, and council members continue to look for opportunities to promote the importance of appointing FPs as well-trained generalists who can contribute to clinical care, supervision, training and clinical governance.^1^ Dr J. Nash and Prof. T. Ras have represented the Academy on the Human Resources of Health planning committee and have been pushing for the creation of FP posts at every district hospital and every Community Health Centre (CHC).
  ■ Community service is under threat, with the National Department of Health (DOH) considering abolishing community service in 2025. We believe that community service officers (CSOs) play a vital role in service delivery in many District Hospitals (DHs) and CHCs around the country, and stopping community service without adequately funding alternative staff would have a major negative effect on service delivery around the country. The Academy has partnered with other members of the rural health alliance to highlight the contributions of CSOs and call for the government to reconsider this decision.
- The voice of SAAFP on important health issues in South Africa:
  ■ The National Health Insurance (NHI) bill was passed by Parliament late in 2023. While we support the principle of Universal Health Coverage (UHC), the current format is unaffordable, does not build on the strengths of the private sector and seems to be unimplementable. We would like to see an effective, affordable healthcare service that provides excellent healthcare to all at a cost that the country can afford and optimally utilises the available healthcare personnel and facilities. We are planning a think tank in May 2024 to clarify the Academy’s position and identify ways we can constructively contribute towards UHC. We would welcome your ideas and input.

On behalf of the new Council, I would like to thank you for your membership and encourage your active participation in the activities of the SAAFP.
